# Populism, euroscepticism, and the populism-euroscepticism nexus

**DOI:** 10.12688/openreseurope.23109.1

**Published:** 2026-03-11

**Authors:** Marta Lorimer, Juan Roch, Stijn van Kessel

**Affiliations:** 1 Politics and International Relations, Cardiff University School of Law and Politics, Cardiff, Wales, UK; 2 Universidad Autonoma de Madrid, Madrid, Community of Madrid, Spain; 3 Queen Mary University of London, London, England, UK

**Keywords:** Populism, euroscepticism, conceptual framework, left-wing populism, right-wing populism, opposition to European Union, European Union

## Abstract

Populism and Euroscepticism have emerged as two of the most salient and contested phenomena in contemporary European politics. This review article develops a conceptual framework for analysing the populism–Euroscepticism nexus within CA23102 ‘Linking euroscepticism and populism: causes and consequences’ (EUPopLink). The article reviews existing literature on populism and Euroscepticism, and discusses the link between the two concepts. It distinguishes three main forms of populism (right-ring populism, left-wing populism, and valence populism) and identifies variations in Euroscepticism based on the object of opposition, the intensity and consistency of opposition and the nature of opposition. It then theoretically explores the nexus between the two concepts. The article concludes by presenting the data collection strategy for the country reports produced by EUPopLink’s Working Group 1. The link between populism and Euroscepticism requires further exploration. The article provides the solid theoretical foundation for such an analysis.

## I. Introduction: Linking populism and euroscepticism

Populism and Euroscepticism are among the salient political phenomena of our time. The COST Action project ‘Linking euroscepticism and populism: causes and consequences’ (EUPopLink) is designed to interrogate in detail the link between the two concepts. Its main objective is to ‘analyse the interplay between Euroscepticism and populism, to identify its causes and consequences and how the negative consequences can be mitigated, using and combining existing datasets and exploring both the supply and the demand side of electoral competition’ (for more information on the project, see Andreadis and Vasilopoulou, 2026).

Solid research requires the establishment of strong conceptual foundations, a task assigned to Working Group 1 (WG1): Conceptual Framework for Populism and Euroscepticism. As part of this Working Group, we developed a conceptual framework defining EUPopLink’s key terms, with a focus on the supply-side of populism and Euroscepticism. Additionally, we coordinated the collection of country reports on populism and Euroscepticism in 40 European countries. This document presents the project’s key definitions, the connections between populism and Euroscepticism, as well as WG1's data collection strategy.

## II. EUPopLink’s key concepts

EUPopLink explores the connection between two core concepts: the concept of populism, and that of Euroscepticism. Both are contested, requiring the adoption of transparent and shared definitions.

### Populism

The use of the term populism remains haphazard in everyday language, but also in academic publications. One key problem remains the conflation of the term with adjacent concepts such as nativism (a xenophobic form of nationalism) or authoritarianism ( De Cleen et al., 2018; Rooduijn, 2019). The academic debate on the concept is nevertheless well-developed and there appears to be a broad consensus that populism entails a normative distinction between the ‘people’ and the out-of-touch or corrupt ‘elites’ ( Canovan, 1999; Taggart, 2000). According to the populist worldview, political elites are, at best, unresponsive to popular demands and, at worst, consciously scheming against ordinary citizens in an effort to further their own self-interest. Populism proposes that politicians should instead respect popular sovereignty, and that political decisions should be taken with the interest of ‘the people’ in mind.

There are still quite important distinctions between key approaches to populism (see Moffitt, 2020 for a systematic overview and comparison). The ‘ideational’ approach is currently dominant amongst political scientists, who note the usefulness of this approach for empirical application (see Hawkins et al. 2018). The guiding definition for this approach is Cas Mudde’s ( Mudde, 2004: 543), which sees populism as ‘an ideology that considers society to be ultimately separated into two homogeneous and antagonistic groups, “the pure people” versus “the corrupt elite”, and which argues that politics should be an expression of the *volonté générale* (general will) of the people’. Mudde argues populism can be seen as a ‘thin-centred’ ideology that makes claims about the (real and desired) relationship between elites and the people, but lacks a concrete policy agenda ( Freeden, 2017). Populism essentially needs ‘host ideologies’ to be added for it to mobilise in practice. For instance, nativism and authoritarianism combine with populism in the ‘exclusionary’ populist radical right (PRR) category ( Mudde, 2007), while populism can merge with socialist values in more ‘inclusionary’ left-wing forms of populism ( Mudde and Rovira Kaltwasser, 2013).

Many scholars focusing on this latter form of progressive and inclusionary populism – which lacks the nativist and xenophobic character of the PRR – subscribe to a different approach to populism inspired by the poststructuralist work of Ernesto Laclau (2005), that can be labelled as the ‘discursive’ approach (e.g., Mouffe, 2018; Stavrakakis and Katsambekis, 2014). For them, populism is essentially a logic of political mobilisation centred on a dichotomy between the people and (those in) power. Key to the populist form of mobilisation is the bringing together of a variety of societal demands and identities through the construction of a so-called ‘chain of equivalence’, which links together diverse constituencies that share a grievance with power holders. The idea of the chain of equivalence contrasts with the element of Mudde’s (2004) definition that refers to the assumed homogeneity of both the ‘elite’ and ‘people’ categories. According to Mudde, populism is not only anti-elitist, but also anti-pluralistic: populists subscribe to the idea of a ‘general will’ and a ‘homogeneous people’,and thus deny or ignore the existence of inherent socio-economic and socio-cultural divisions within society. By contrast, the discursive approach conceives populism as a political logic through which demands are articulated within a ‘heterogeneous and internally differentiated people’. From this perspective, populism does not construct ‘the people’ as homogeneous; rather, it temporarily unifies diverse groups and demands without erasing or undermining their particular identities ( Stavrakakis, 2026: 2).

This key difference between the ideational and discursive approaches has important implications for more normative appraisals of the concept. If populism is inherently anti-pluralist, and denies societal heterogeneity, it is an illiberal force that many scholars therefore see as a threat to liberal democracy, or even democracy as such ( Müller, 2016). Furthermore, if Mudde’s definition is strictly followed, it is questionable whether parties with a culturally progressive profile that acknowledge cultural diversity – essentially many of the left-wing populists – can genuinely be populist in the first place. ^1^ The idea of the ‘chain of equivalence’, conversely, allows for a more inclusive conception of ‘the people’, and scholars following the ‘Laclauian’ approach often consider populism to be a potential emancipatory force when it is employed by the ‘left’ (e.g., Mouffe, 2018; Stavrakakis and Katsambekis, 2014). In this perspective, populism functions as a means to unify and strengthen partial identities and demands that have previously been ignored by institutionalised politics. As such, it contributes to incorporating underrepresented or marginalised groups and identities into the political sphere.

Yet there are also important overlaps between the ideational and the discursive approaches. They both start out from the idea that the two central categories to populism (‘the people’ and ‘the elites’) are empty vessels that need to be given a more concrete interpretation (depending on political context and ‘host ideology’). As far as ‘the people’ are concerned, the PRR refers to their ethno-cultural characteristics, while left-wing populists tend to define the category with reference to economic status (i.e. the ‘people’ become the underprivileged and economically marginalised, versus the privileged elite minority that holds power). The ‘people’ and ‘elite’ are thus given a meaning that is consistent with the broader political project of populist actors ( De Cleen et al., 2018).

While the people vs elite dichotomy is a broadly accepted key aspect of populism, there are various other approaches that see populism as yet a different type of phenomenon, such as a ‘discursive frame’ ( Aslanidis, 2024), a (communication) ‘style’ ( de Vreese et al., 2018; Moffitt, 2017; Walgrave and Swert, 2004) or a ‘strategy’ ( Weyland, 2001). Moffitt’s stylistic approach and Weyland’s strategic approaches focus, respectively, on the performative features of populist leadership (including ‘bad manners’) or the way in which a personalistic leader mobilises support. Such approaches to populism are therefore particularly (or even exclusively) suitable for analysing the political ‘supply side’ that covers the behavioural and organisational characteristics of politicians and political parties. Many scholars following the ‘ideational approach’, on the other hand, have argued that populism can also exist as an attitude held by citizens on the political ‘demand side’, and showed that these attitudes also matter in terms of explaining support for populist actors ( Akkerman et al., 2014; Van Hauwaert and van Kessel, 2018).

For EUPopLink, we need to outline the concept’s key attributes in order to study it in a meaningful and systematic way. It is therefore necessary to take a position in key conceptual debates. For the purpose of this project, populism will be treated as a set of jointly necessary ideas or claims that revolve around the distinction between ‘people’ and ‘elite’. Put simply, a phenomenon is only populist when it includes *both* elements of anti-elitism *and* an appeal to the ideal of popular sovereignty. From this it follows that not all ‘anti-establishment’ actors are also populist, given that not all of them present themselves as the ‘voice of the people’ – they may instead propose to replace the current elite with a new elite (e.g. a communist vanguard party that makes the people aware of their ‘real interests’).

This conceptualisation is essentially in line with the discursive and ideational approach, although it is still a matter of debate whether populism constitutes a genuine ‘ideology’ (thin-centred or not) or whether it is perhaps more useful to consider it as a ‘discursive frame’ that provides a certain interpretation of reality ( Aslanidis, 2024). Given that certain political parties express populism frequently and more or less consistently (while others do not), it also makes sense to identify a group of so-called ‘populist parties’ (see van Kessel, 2014). This does not preclude the possibility that populist claims can be made (whether for strategic reasons or not) by ‘non-populists’ as well. Populism can therefore both be used as a ‘classifier’ (to refer to a specific type of politicians and parties) as well as a type of discourse that can be used sporadically and temporarily. We also concur with the notion that populism can be measured at the political supply side (i.e. expressed by political actors) as well as the political demand side (i.e. as an attitude of citizens).

It is also crucial to take a clear position on the question of whether populism is inherently anti-pluralistic, and therefore anti-liberal. While the easiest way to define ‘the people’ is arguably by pointing out who does not belong to this category, in our conception populism does not strictly need to treat ‘the people’ as a homogeneous entity, seeing the people’s will as singular and indivisible. If this were the case, the concept becomes unsuitable for presumed (left-wing) populist actors (such as Podemos and Syriza) – and it would be questionable if populism can be ‘left-wing’ at all. Not all forms of populism are xenophobic, and the construction of ‘the people’ can, to a great extent, be inclusionary. In terms of more wide-ranging implications of populism, rather than seeing populism as a phenomenon that *either* threatens *or* revitalises democracy, we can subscribe to Margaret Canovan’s (1999) analysis of populism as a phenomenon that is inherently linked to democracy, and that feeds on the tensions between the promises of democracy as an ideal and the realities of democratic government in practice (or: the ‘redemptive’ and ‘pragmatic’ faces of democracy).

Based on the above considerations, our working definition of populism is as follows: **a political discourse centred on an antagonism between ‘people’ and ‘elites’ that expresses support for popular sovereignty versus unresponsive or corrupt elite rule**. In the European context, populism as expressed consistently by a certain set of political actors can furthermore be analysed using the following subcategories:

1) **The populist radical right (PRR):** Political actors that combine populism with a) nativism (the idea that members of the native group should be prioritised and that non-native elements are fundamentally threatening), and b) authoritarianism (adhering to the belief in a strictly ordered society with inherent natural inequalities) (see Mudde, 2007);
2) **Left-wing populists:** Political actors that express populism and ‘reject the underlying socio-economic structure of contemporary capitalism’ and ‘advocate for alternative economic and power structures involving a major redistribution of resources from existing political elites’ ( March, 2011: 8).
3) **Valence populists:** Political actors that express populism and ‘that predominantly, if not exclusively, compete by focusing on nonpositional issues such as the fight against corruption, increased transparency, democratic reform and moral integrity, while emphasizing anti-establishment motives’ ( Zulianello, 2020; Zulianello and Larsen, 2024).

In contemporary Europe, the PRR is electorally outflanking left-wing forms of populism, which previously experienced success mainly in Spain (Podemos) and Greece (SYRIZA) in the wake of the financial and economic crises of ca. 2008–2015. The PRR has witnessed a steady increase/consolidation of its support over the past decades, and a more recent expansion to countries (e.g. Germany, Sweden, Spain, Portugal) where it was previously unsuccessful. ‘Valence populism’ - arguably a less tangible category – has been particularly widespread in Central and Eastern Europe as a characteristic of parties that expressed dissatisfaction with the transition process from communism to liberal democracy and capitalism, and that typically lamented the corruption of post-communist political elites. The Five Star Movement (M5S) in Italy can also be seen as a case of ‘valence populism’ given its key focus on the quality of government and original aim to revolutionise the political process. It is important to emphasise that valence populists obviously do adopt specific positions on concrete political issues related to the economy, migration, the environment etc.; ‘however, their policy stances are primarily informed by an unadulterated conception of populism (with other ideological elements, if any, playing a marginal or secondary role), and are therefore flexible, free-floating and, often, inconsistent’ ( Zulianello, 2020: 332). It is possible, however, that actors transition from one to another type of populism across time; a case in point is the Hungarian Fidesz which moved to PRR territory in the past few decades.

### Euroscepticism

The term Euroscepticism first came into use in the mid-1980s, when journalists and politicians started using it to refer to British Conservative MPs who were sceptical about the path of European integration ( Leruth et al., 2018: 14; Spiering, 2004). Since then, the term has been appropriated by researchers in European politics, and developed into a distinct field of study analysing opposition to the European Union amongst both elites and general publics.

In its first usages (and to some extent, to this day), the term Euroscepticism lacked precision, combining as it did two terms lacking specificity. The ‘Euro’ part, taken to refer to the European Union, remained difficult to pin down because it was not clear what part of the European project one would have to object to be defined a Eurosceptic. This was compounded by the complexity of the EU itself, an unfinished organisation in constant development, with shifting geographical and political boundaries. The ‘sceptic’ part posed even more problems, to the extent that it seemed to potentially cover a variety of positions going from friendly constructive criticism to outright rejection of the European project as a whole. Ultimately, what made Euroscepticism difficult to pin down is the fact that it was (and still is), a negative construction. As Benjamin Leruth, Nicolas Startin and Simon Usherwood put it, Euroscepticism, ‘refers to opposition to some aspect of European integration, the very vagueness of which merely makes the point that it risks being everything and nothing. The concept does not say anything about why that opposition exists, what form it should take, to what it should apply, nor to what end’ ( Leruth et al., 2018: 4).

To bring precision to the term, several definitions have emerged over the years. The first definition of party-based Euroscepticism was provided by Paul Taggart in 1998, when he defined it as ‘the idea of contingent or qualified opposition, as well as incorporating outright and unqualified opposition to the process of European Integration’ ( Taggart, 1998: 366). In later work with Aleks Szczerbiak, they further distinguished between ‘hard’ and ‘soft’ Euroscepticism ( Szczerbiak and Taggart, 2008a, 2008b; Taggart and Szczerbiak, 2002). In its final formulation, Szczerbiak and Taggart’s definition considers ‘hard’ Euroscepticism to exist when ‘there is a principled opposition to the EU and European integration and therefore can be seen in parties who think that their countries should withdraw from membership, or whose policies towards the EU are tantamount to being opposed to the whole project of European integration as it is currently conceived’ ( Szczerbiak and Taggart, 2008a: 7–8). ‘Soft’ Euroscepticism, instead, ‘is where there is not a principled objection to European integration or EU membership but where concerns on one (or a number) of policy areas lead to the expression of qualified opposition to the EU, or where there is a sense that ‘national interest’ is currently at odds with the EU’s trajectory’ ( Szczerbiak and Taggart, 2008a: 7–8).

Taggart and Szczerbiak’s definition has been very influential and has the advantage of being both parsimonious and able to capture some of the variation that exists in Euroscepticism as a phenomenon. However, it remains limited concerning what is being criticized and how. This is particularly true for the category of ‘soft’ Euroscepticism which, while more specific than in the original formulation of 2002, remains somewhat broad (although ‘hard’ Euroscepticism has also come under scrutiny, e.g., Havlík and Hloušek, 2025).

With the shortcomings of the original definition in mind, others have sought to develop fine-grained categorisations of Euroscepticism that would be better able to capture the phenomenon’s variation. Cas Mudde and Petr Kopecky were amongst the first to take this path and bring attention to what, exactly, it is that ‘Eurosceptics’ criticise. Drawing on David Easton’s seminal distinction regarding the support for political regimes, their work focused on a two- dimensional conceptualization between diffuse and specific support for European integration. More specifically, they define diffuse support as ‘support for the general ideas of European integration’, while specific EU support is defined as ‘support for the general practice of European integration’ ( Kopecky and Mudde, 2002). Based on this framework of analysis, they offer a typology of party positions on Europe based on four subcategories. These are: (1) the ‘Euroenthusiasts’ who support the idea of European integration and are in favour of its institutionalisation; (2) the ‘Eurosceptics’ who combine pro-EU stances such as the support of European integration, but at the same time tend to adopt a more pessimistic view about its current form; (3) the ‘Eurorejects’ who are against both the idea of the EU and the process of European integration and finally, (4) the ‘Europragmatists’ who reject the principle of European integration, but support the EU in its current form on a more utilitarian basis ( Kopecky and Mudde, 2002).

In a similar spirit, Peter Mair (2007) suggested that one should distinguish between ‘policy’ Euroscepticism and ‘polity’ Euroscepticism. ‘Policy’ Euroscepticism captures an expression of disagreement with particular EU policies such as the Single Market, or the Common Agricultural Policy. ‘Polity’ Euroscepticism refers to a more fundamental opposition to the EU as a political system and as a consequence, against EU membership as well (for an analysis see also De Vries, 2018; Verney, 2017). For her part, in work on far-right Euroscepticism, Sofia Vasilopoulou (2018) proposes to distinguish between party positions on the principle, practice and future of European integration, thus bringing a future-orientation to previous definitions. Her typology presents three main categories: rejectionist Eurosceptics, who oppose the principle of European cooperation, the practice of EU policy and any future integration project; conditional Eurosceptics, who are in favour of European cooperation, but oppose the EU’s current form and are against more in-depth cooperation; and compromising Eurosceptics, who accept both the principle and current practice of the EU, but are averse to integrating further.

Other definitions focus more on how intensely the EU is being critiqued. This is the case of the definition advanced by Chris Flood and Simon Usherwood (2005), who seek to build a classification that will better capture degrees of support for, or opposition to EU integration. In their approach, they introduce six categories, with an optional seventh, to capture positions on both the EU project in general and specific aspects of it. The categories are, from least supportive to most supportive: rejectionist (outright refusal of integration and opposition to participation); revisionist (support for return to a previous phase of integration); minimalist (accepting of the status quo, but wanting to go no further in integration); neutral (no position); gradualist (accepting advances of integration, so long as they are slow and piecemeal); reformist (supporting advances in integration, subject to remedying existing shortcomings); and maximalist (pushing integration forwards towards the realization of one’s preferred outcome). In a similar vein, Ray (1999) asked country experts about parties’ positions on European integration as placed on a scale, how important these parties considered the issue, and the degree of internal disagreement over the issue. Later studies have built on this work—most notably the Chapel Hill Expert Survey ( Rooduijn and van Kessel, 2019), which provides up-to-date longitudinal data on party positions on a variety of issues, including positions on the EU.

The definitions of Euroscepticism discussed so far do not capture one last element of variation in Euroscepticism, that is, the variation in terms of the substantive content of Eurosceptic critiques. Actors belonging to different ideological traditions will be Eurosceptic for different reasons, something that is particularly noticeable amongst radical parties of the left and of the right. Scholars have noted that while parties at the ideological fringes tend to share Eurosceptic positions, the radical left and right express distinctive arguments against the EU ( Conti and Memoli, 2011) and use different ‘frames’ in opposing European integration ( Helbling et al., 2010; Pirro et al., 2018). The radical right typically portrays the EU as a project that threatens the sovereignty of the native people and, through the opening of borders, the cultural homogeneity of nations. Radical left parties typically describe European integration as a neoliberal project that encourages a ‘race to the bottom’ in terms of welfare entitlements and working conditions.

While the above definitions suggest that Euroscepticism is a somewhat static and clear-cut phenomenon, it is worth noting that it is in fact a more flexible construct than many of these typologies capture ( Roch, 2025). Parties can modulate their positions based on evolving political contexts, switching from supporting EU integration to opposing it and vice versa. This was the case of some populist radical right parties such as the French National Rally, which moved from being relatively positive about the EU to being distinctly negative about European integration ( Lorimer, 2021, 2024; Mudde, 2007). Parties can also adopt deliberately ‘equivocal’ stances, alternating between soft and hard Euroscepticism, to achieve key strategic goals ( Heinisch et al., 2021).

Furthermore, even though the terms are used interchangeably in practice, ‘Europe’ and the EU are not the same, and political actors can discursively exploit that distinction. Parties of the populist radical right, for instance, may depict ‘Europe’ as the birthplace of Western civilisation, emphasising the common cultural heritage of the various European nations. Such a notion is helpful in constructing the ‘other’ as well as the identity of ‘the people’ these parties appeal to. A positive conception of a shared European identity can be accompanied by critical attitudes towards the actions of the EU as an organisation and towards the actions of member states’ governments. Expressing ‘Euro-ambivalence’, far-right parties can claim allegiance to ‘Europe’, helping them to shed an image of narrow-mindedness and parochialism, while denouncing the EU, or at least its current direction, as a political project ( Lorimer, 2021, 2024). The idea of Europe being a malleable concept helps us in understanding why populist (and other ‘outsider’) parties’ ideas and positions on the issue of European integration are not that clear-cut.

Once the prerogative of marginal movements, Euroscepticism has increasingly moved in the political mainstream ( Taggart and Szczerbiak, 2013). The era of the polycrisis has facilitated this process, showing as it did that not all was well with EU integration. Euroscepticism has thus been adopted by mainstream actors, who have become increasingly critical of the process of EU integration themselves. Additionally, the progressive normalisation of radical movements has also led to Euroscepticism becoming a less marginal phenomenon. One should, however, also not over-state its importance, to the extent that political parties, for the most part, still give the EU issue less salience than to other issues ( Braun and Schmitt, 2020; Hix and Cunningham, 2026; Marks and Steenbergen, 2004).

Based on the above considerations, we follow Paul Taggart in defining Euroscepticism as ‘the idea of contingent or qualified opposition, as well as incorporating outright and unqualified opposition to the process of European Integration’ ( Taggart, 1998: 366) and specify three key aspects that help capture variation within this broad definition:

• **Object of opposition:** This element captures what aspect of EU integration is being critiqued and touches upon both the ‘polity’ and ‘policy’ distinction (is a party critiquing the institutional design of the EU or specific policy measures?), as well as the principle, practice and future of EU integration (is its opposition centred on the idea of European collaboration, the specific form it takes in the EU, or its future direction?)
• **Intensity and consistency of opposition:** This aspect captures the strength of the critique. It helps distinguish between ‘hard’ or ‘soft’ opposition (how intense is the party’s opposition to EU integration?), as well as identify how consistently the EU is being critiqued (to what extent are positions consistently held?). As such, it can also help address elements of ambivalence in party position.
• **Nature of opposition:** This element captures the substantive content of opposition to the EU (for what reasons is the EU opposed?). Rather than focusing on what is being critiqued and how strongly, it considers why the EU is being criticised and the specific nature of the arguments that are brought against it. The nature of opposition will usually depend on a party’s broader ideological positioning (e.g., left-wing or right-wing).

Although these categories are analytically separate, there may be overlaps between them. Taken together, they provide a comprehensive overview of party-based Euroscepticism.

## III. The populism-Euroscepticism nexus

Acknowledging the heterogeneity of party positions and the evolving nature of EU critique, it remains possible to identify potential connections between populist discourse and Euroscepticism. EUPopLink develops theoretical expectations that are sensitive to the diversity, complexity, and fluidity of both populism and Euroscepticism, as previously defined. We map the fundamental linkages between populism and Euroscepticism. Subsequently, we explore how specific variants of populist discourse intertwine with opposition to the EU or critiques of its structure and policies.

When we consider the essential characteristics of populism, it comes as no surprise that parties with a populist profile typically depict the EU as an elite-driven organisation that operates far removed from ordinary citizens and whose complex decision-making processes stand in the way of implementing the popular will ( Canovan, 1999: 199; Rooduijn and van Kessel, 2019). Yet it has also become clear that most populists (on both the radical left and right) are by no means ‘hard Eurosceptic’; that is, principally opposed to every form of European integration ( Szczerbiak and Taggart, 2024). The tumultuous Brexit process appears to have dampened the enthusiasm of vocal Eurosceptics for their country to leave the Union ( Martini and Walter, 2024; van Kessel et al., 2020). More fundamentally, populist parties do not usually rule out all forms of European cooperation. PRR parties typically advocate collaboration in the form of a ‘Europe of sovereign nations’. They may look positively upon the EU as an economic community as long as it benefits the prosperity of their nation, or see a role for the EU in dealing with ‘outside threats’ they identify, not least immigration from non-Western countries ( Lorimer, 2021). Parties and social movements on the radical left – which in fact often desire *more* transnational cooperation – also call for ‘another Europe’ that prioritises economic and social justice, as well as democratisation ( della Porta, 2020).

Previous studies have thus already pointed out that parties placing themselves outside of the so-called mainstream are not united in their opposition towards Europe – be it ‘far-right’ ( Vasilopoulou, 2018), ‘populist’ ( Pirro et al., 2018) or ‘anti-establishment’ ( Szczerbiak and Taggart, 2024) parties. Yet there is still a tendency to treat the connection between radical populist politics and Euroscepticism as a given. Gaining a more nuanced understanding of the (current) arguments and ideas of various types of (self-styled) ‘political outsiders’ is important to move beyond overly simplistic assumptions. This is even more pertinent in light of the electoral growth and consolidation of the PRR party family at the national and European levels, and these parties becoming part of governing coalitions in an increasing number of countries. The influence PRR parties can potentially exert on the process of European integration has never been larger – although this would presume a reasonably united stance and coherent perspective on European integration on the side of these parties, which are both essentially lacking.

To gain a precise understanding of the populism-Euroscepticism nexus it is important to consider ‘Europe’ as a ‘malleable’ concept – as we have discussed above - that does not necessarily refer to the EU and its institutions ( Lorimer, 2024; Roch, 2025). In its broadest sense, the populist discourse—understood as a framework for constructing ‘the people’ and distinguishing them from “the elites”—exhibits certain affinities with Euroscepticism. This connection becomes particularly evident if the EU is perceived as an elite-driven project ( Beeson and Diez, 2018: 119; Bulmer and Joseph, 2016: 739; Hobolt, 2018: 243). In this context, the populist construction of ‘the people’, rooted in aggregating demands and recognizing popular identities, is often at odds with less directly accountable EU bodies such as the European Central Bank (ECB) or the European Commission. Consequently, populist discourse as such tends to align with a diffuse form of Euroscepticism, not necessarily focusing on the EU polity as a whole but rather on specific policies or institutions undermining the people’s interest as well as perceived democratic deficits within EU institutions.

Anti-establishment rhetoric within populist discourse is often strongly associated with various expressions of Euroscepticism, spanning across the ideological spectrum ( Szczerbiak and Taggart, 2024). For instance, Italy’s League and Five Star Movement used anti-establishment discourse to criticise the EU, particularly during the Eurozone and migration crises. Similarly, the National Rally and France Unbowed in France frame their Euroscepticism as opposition to EU elites, depicting them as part of a global establishment. The appeal to popular sovereignty is also shared by different types of populist actors voicing Euroscepticism, whereby the EU is portrayed as unresponsive to or insulated from popular demands. However, ideological leaning is crucial to determine the substantive content and arguments forming the EU critique of political actors.

When populist discourse is employed by left-wing parties, the EU’s social pillar—articulated through measures like the European Social Charter or redistribution initiatives—tends to be framed positively in contrast to the EU’s more neoliberal components, such as the ECB’s monetary policies or trade agreements like the TTIP ( Oleart, 2021). In critiquing the EU from this perspective, left-wing populist parties often emphasize a vision of social justice, portraying European integration as a neoliberal project that fosters a ‘race to the bottom’ in welfare standards and labour conditions (see De Vries and Edwards, 2009). These left-wing populist movements are typically associated with ‘soft’ or qualified Euroscepticism ( della Porta, 2020), although they may go through different stages in their EU opposition, changing the intensity or scope of their critique. For instance, Podemos in Spain evolved from an initial stage of critique of the EU as a polity to a qualified opposition to specific policies and institutional developments ( Roch, 2021).

A second variant of populism, the populism-nativist discourse, delineates ‘the people’ along nationalistic and identity-based lines ( Lobera and Roch, 2022). This version of populism shares strong affinities with Euroscepticism, particularly as articulated by PRR parties. These tend to be the fiercest critics of the EU: besides voicing populist critique, they lament the loss of national sovereignty, which they consider the result of deeper European integration, and they dislike the opening of borders which is associated with a rise in immigration. The populist-nativist discourse fundamentally opposes forms of European integration that are perceived as undermining national sovereignty. It advocates for a vision of a self-contained and self-sufficient nation, prioritizing national interests over transnational cooperation. Within this conception, the EU appears as a bureaucratic apparatus in opposition to national identity and sovereignty. While these critiques may also target specific policies, populism-nativism ultimately aspires to liberate the nation and its identity from the EU integration project entirely. However, pragmatic political strategies have often tempered these impulses, leading PRR parties to advocate for a minimalistic EU framework or a loose alliance of sovereign states.

Besides the fact that radical populist parties are rarely fully opposed to the idea of European integration, we also see divisions inside Eurosceptic party families. Within the PRR, points of contention are, for example, positions towards Russia or the extent to which the EU should foster free trade. The fact that no single radical right European Parliament group has been formed is partly grounded in diverging national interests and ideological viewpoints, but perhaps more important are strategic choices and presumed reputational consequences of allying with foreign partners ( McDonnell and Werner, 2019). The Alternative for Germany is now shunned by fellow party family members given its extremist tendencies, whereas other PRR parties, such as Brothers of Italy, led by Italian Prime Minister Giorgia Meloni, have been successful in softening their image. Meloni enjoys seemingly cozy and constructive relationships with various European leaders, illustrating the continuing legitimisation and normalisation of the far right ( Mudde, 2019).

Finally, when the populist discourse portrays ‘the people’ as a transnational *demos* ( Moffitt, 2017), the distinction between Europe and the EU—already present in the previously discussed modalities of critique—becomes more pronounced. In this perspective, efforts to construct a European identity for the transnational *demos* often lead to an evaluation of the EU as a flawed integration project. However, alternative visions of transnational identity and international solidarity, grounded in a proto-European identity, are regarded as desirable. In this context, critiques of the EU are typically diffuse, focusing on bureaucratic inefficiencies or detrimental policies that are seen as further alienating EU institutions from the ideal of a bottom-up federation of European peoples.

## IV. Data collection

To map the linkages of populism and euroscepticism in Europe, Working Group 1 coordinated the submission of 40 country reports on populism and Euroscepticism in Europe. The countries cover EU member states, candidate countries, and European countries maintaining a close affiliation with the European Union. To identify authors, we first invited members of the WG1 to indicate which country(-ies) they had relevant expertise on. Based on their responses, we assigned them the task of writing a country report on their selected country of expertise. As not all countries were covered, we sought additional country experts for missing countries. Authors were provided with a series of guidelines to follow when researching and writing their reports. ^2^ Our focus was on secondary research, rather than on original primary research. All authors submitted their reports in the summer of 2025. The reports were then reviewed by EUPopLink Core Group members, and authors invited to revise and resubmit their reports to their assigned reviewer. Once all corrections were approved, authors were invited to submit their case studies to the Open Research Europe (ORE) Platform. Completed reports are available to view, with remaining ones expected to become available on a rolling basis. ^3^

## V. Conclusion

In this paper, we have summarised existing literature on populism, Euroscepticism and their linkage. We put forward three main definitions of populism: radical right-wing populism, left-wing populism and valence populism, and distinguished between opposition to the EU based on which aspect of the EU is being critiqued, how intensely and consistently this is done, and what perspective opposition is articulated from. We concluded by addressing the links between populism and Eurosceptcisism, and discussing how they were studied in the EUPopLink case study collection available on the Open Research Europe platform.

## Ethics and consent

Ethical approval and consent were not required.

## Data Availability

No data are associated with this article.

## Footnotes

^1^
Notably, however, many scholars in the ideational tradition still consider socially liberal parties such as Podemos in Spain and SYRIZA in Greece as instances of ‘left-wing’ populism. Furthermore, Mudde and Rovira Kaltwasser (2013) claim it is possible to identify an ‘inclusionary’ form of populism based on Mudde’s definition.

^2^
The complete guidelines are available at the following address: https://eupoplink.eu/node/17

^3^
Access the full collection here: https://open-research-europe.ec.europa.eu/collections/eupoplink/about

## References

[ref1] Akkerman A Mudde C Zaslove A : How Populist Are the People? Measuring Populist Attitudes in Voters. *Comp. Pol. Stud.* 2014;47(9):1324–1353. 10.1177/0010414013512600

[ref2] Andreadis I Vasilopoulou S : The populism-euroscepticism nexus in a contested Europe: The EUPopLink COST Action: [version 1; peer review: awaiting peer review]. *Open Research Europe.* 2026;6(27). 10.12688/openreseurope.22680.1 PMC12910195 41710161

[ref3] Aslanidis P : Populist Mobilization. *Oxford scholarship online.* Oxford: Oxford University Press;2024.

[ref4] Beeson M Diez T : Responding to crises: Europe and Southeast Asia. *Asia Eur J.* 2018;16(2):115–124. Berlin/Heidelberg: Springer Berlin Heidelberg.

[ref5] Braun D Schmitt H : Different emphases, same positions? The election manifestos of political parties in the EU multilevel electoral system compared. *Party Polit.* 2020;26(5):640–650. London, England: SAGE Publications.

[ref6] Bulmer S Joseph J : European integration in crisis? Of supranational integration, hegemonic projects and domestic politics. *Eur. J. Int. Rel.* 2016;22(4):725–748. London, England: SAGE Publications. 10.1177/1354066115612558 PMC5898385 29708125

[ref7] Canovan M : Trust the People! Populism and the Two Faces of Democracy. *Political Studies.* 1999;47:2–16. 10.1111/1467-9248.00184

[ref8] Conti N Memoli V : The multi-faceted nature of party-based Euroscepticism. *Acta Polit.* 2011;47(2):91–112.

[ref9] De Cleen B Glynos J Mondon A : Critical research on populism: Nine rules of engagement. *Organization (London, England).* 2018;25(5):649–661. London, England: SAGE Publications.

[ref10] Vreese CH de Esser F Aalberg T : Populism as an Expression of Political Communication Content and Style: A New Perspective. *The international journal of press/politics.* 2018;23(4):423–438. Los Angeles, CA: SAGE Publications. 30886670 10.1177/1940161218790035 PMC6380726

[ref11] De Vries CE : *Euroscepticism and the Future of European Integration.* Oxford: Oxford University Press;2018.

[ref12] De Vries CE Edwards EE : Taking Europe To Its Extremes: Extremist Parties and Public Euroscepticism. *Party Polit.* 2009;15(1):5–28.

[ref13] della Porta D : Europeanisation from below: Still time for another Europe? Introduction to the special issue of the European Journal of Cultural and Political Sociology. *Eur. J. Cult. Polit. Sociol.* 2020;7(3):225–241. Routledge.

[ref14] Flood C Usherwood SM : Positions, Dispositions, Transitions: A model of group alignment on EU integration. *Paper presented at the 55th Annual Conference of the Political Studies Association.* University of Leeds;2005 5–7 April 2005. Reference Source

[ref15] Freeden M : After the Brexit referendum: Revisiting populism as an ideology. *Journal of Political Ideologies.* 2017;22:1–11. 10.1080/13569317.2016.1260813

[ref16] Havlík V Hloušek V : Where Have All the ‘Exiters’ Gone? Contextualising the Concept of Hard Euroscepticism. *J. Common Mark. Stud.* 2025;63(2):351–368. Oxford: Blackwell Publishing Ltd.

[ref66] Hawkins KA Carlin RE Littvay L : editors. *The Ideational Approach to Populism: Concept, Theory, and Analysis.* Routledge; 1st ed. 2018. 10.4324/9781315196923

[ref17] Heinisch R McDonnell D Werner A : Equivocal Euroscepticism: How Populist Radical Right Parties Can Have Their EU Cake and Eat It. *J. Common Mark. Stud.* 2021;59:189–205. 10.1111/jcms.13055

[ref18] Helbling M Hoeglinger D Wüest B : How political parties frame European integration. *Eur. J. Polit. Res.* 2010;49(4):495–521. Oxford, UK: Blackwell Publishing Ltd.

[ref19] Hix S Cunningham K : Still second-order national elections? Evaluating the classic model after the 2024 European elections. *West Eur. Polit.* 2026;49(1):243–264. London: Routledge.

[ref20] Hobolt SB : The crisis of legitimacy of European institutions. *Europe’s Crises.* Polity Press;2018;243–267

[ref21] Kopecky P Mudde C : The Two Sides of Euroscepticism: Party Positions on European Integration in East Central Europe. *Eur. Union Polit.* 2002;3(3):297–326.

[ref22] Laclau E : Populism: What’s in a Name. *Populism and the Mirror of Democracy.* London: Verso;2005;32–49.

[ref23] Leruth B Startin N Usherwood SM : *Routledge Handbook of Euroscepticism.* London: Routledge;2018.

[ref24] Lobera J Roch J : A nationalist party with non-nationalistic voters? Discussing the limits of nationalism in party categorisation. *Nations Natl.* 2022;28(2):539–556. John Wiley & Sons, Ltd.

[ref25] Lorimer M : What do they talk about when they talk about Europe? Euro-ambivalence in far right ideology. *Ethn. Racial Stud.* 2021;44(11):2016–2033. Taylor & Francis.

[ref26] Lorimer M : *Europe as Ideological Resource: European Integration and Far Right Legitimation in France and Italy.* Oxford: Oxford University Press;2024.

[ref27] Mair P : Political Opposition and the European Union. *Gov. Oppos.* 2007;42(1):1–17. Blackwell Publishing. 10.1111/j.1477-7053.2007.00209.x

[ref28] March L : *Radical Left Parties in Europe.* London: Routledge;2011.

[ref29] Marks G Steenbergen MR : *European Integration and Political Conflict. European Integration & Political Conflict.* Cambridge: Cambridge University Press;2004.

[ref30] Martini M Walter S : Learning from precedent: how the British Brexit experience shapes nationalist rhetoric outside the UK. *J. Eur. Publ. Policy.* 2024;31(5):1231–1258. England: Routledge. 10.1080/13501763.2023.2176530 PMC11166053 38868721

[ref31] McDonnell D Werner A : *International Populism: The Radical Right in the European Parliament.* London: Hurst;2019.

[ref32] Moffitt B : *The Global Rise of Populism: Performance, Political Style, and Representation.* Stanford: Stanford University Press;2017.

[ref33] Moffitt B : *Populism.* Cambridge: Polity Press;2020.

[ref34] Mouffe C : *For a Left Populism.* London: Verso;2018.

[ref35] Mudde C : The Populist Zeitgeist. *Gov. Oppos.* 2004;39(3):541–563.

[ref36] Mudde C : *Populist Radical Right Parties in Europe.* Cambridge: Cambridge University Press;2007.

[ref37] Mudde C : *The Far Right Today.* Cambridge, UK: Polity;2019.

[ref38] Mudde C Rovira Kaltwasser C : Exclusionary vs. Inclusionary Populism: Comparing Contemporary Europe and Latin America. *Gov. Oppos.* 2013;48(2):147. Cambridge.

[ref39] Müller J-W : *What Is Populism?.* Philadelphia: University of Pennsylvania Press;2016. 10.9783/9780812293784

[ref40] Oleart A : *Framing TTIP in the European Public Spheres: Towards an Empowering Dissensus for EU Integration.* Cham, Switzerland: Palgrave Macmillan;2021.

[ref41] Pirro ALP Taggart P Kessel S van : The populist politics of Euroscepticism in times of crisis: Comparative conclusions. *Politics (Manchester, England).* 2018;38(3):378–390. London, England: SAGE Publications.

[ref42] Ray L : Measuring party orientations towards European integration: results from an expert survey. *Eur. J. Polit. Res.* 1999;36(2):283–306. Oxford, UK: Blackwell Publishing Ltd.

[ref43] Roch J : Friends or foes? Europe and ‘the people’ in the representations of populist parties. *Politics (Manchester, England).* 2021;41(2):224–239. London, England: SAGE Publications.

[ref44] Roch J : *The Populism-Euroscepticism Nexus: A Discursive Comparative Analysis of Germany and Spain.* London: Routledge; 1st ed. 2025. Reference Source

[ref45] Rooduijn M : State of the field: How to study populism and adjacent topics? A plea for both more and less focus. *Eur. J. Polit. Res.* 2019;58(1):362–372. Oxford: Wiley Subscription Services, Inc.

[ref46] Rooduijn M Kessel S van : Populism and Euroskepticism in the European Union. Oxford: Oxford University Press;2019.

[ref47] Spiering M : British Euroscepticism. *Euroscepticism: Party Politics, National Identity and European Integration.* Amsterdam and new York: Rodopi;2004;127–149.

[ref48] Stavrakakis Y : Populism, Populism Research, and Their Discontents: Strategies of Interpretation from a Psychosocial Perspective. *Curr. Anthropol.* 2026;S000–S000.

[ref49] Stavrakakis Y Katsambekis G : Left-wing populism in the European periphery: The case of SYRIZA. *Journal of Political Ideologies.* 2014;19(2):119–142. 10.1080/13569317.2014.909266

[ref50] Szczerbiak A Taggart P : *Opposing Europe?: The Comparative Party Politics of Euroscepticism. Volume I: Case Studies and Country Surveys.* Oxford: Oxford University Press;2008a.

[ref51] Szczerbiak A Taggart P : *Opposing Europe?: The Comparative Party Politics of Euroscepticism. Volume II: Comparative and Theoretical Perspectives.* Oxford: Oxford University Press;2008b .

[ref52] Szczerbiak A Taggart P : Euroscepticism and anti-establishment parties in Europe. *J. Eur. Integr.* 2024;46(8):1171–1191. Abingdon: Routledge.

[ref53] Taggart P : A touchstone of dissent: Euroscepticism in contemporary Western European party systems. *Eur. J. Polit. Res.* 1998;33(3):363–388. Dordrecht.

[ref54] Taggart P : *Populism. Concepts in the Social Sciences.* Buckingham: Open University Press;2000.

[ref55] Taggart P Szczerbiak A : The Party Politics of Euroscepticism in EU Member and Candidate States. SEI Working Papers 51. 2002.

[ref56] Taggart P Szczerbiak A : Coming in from the Cold? Euroscepticism, Government Participation and Party Positions on Europe. *J. Common Mark. Stud.* 2013;51(1):17–37. Oxford, UK: Blackwell Publishing Ltd.

[ref57] Van Hauwaert SM Kessel S van : Beyond protest and discontent: A cross-national analysis of the effect of populist attitudes and issue positions on populist party support. *Eur. J. Polit. Res.* 2018;57(1):68–92. Oxford: Wiley Subscription Services, Inc.

[ref58] Kessel S van : The populist cat-dog: applying the concept of populism to contemporary European party systems. *J. Polit. Ideol.* 2014;19(1):99–118. Abingdon: Routledge.

[ref59] Kessel S van Chelotti N Drake H : Eager to leave? Populist radical right parties’ responses to the UK’s Brexit vote. *Br. J. Polit. Int. Rel.* 2020;22(1):65–84. London, England: SAGE Publications.

[ref60] Vasilopoulou S : *Far Right Parties and Euroscepticism: Patterns of Opposition.* London; New York: Rowman & Littlefield International;2018.

[ref61] Verney S : Losing Loyalty: The rise of polity Euroscepticism in Southern Europe. *The Routledge Handbook of Euroscepticism.* Routledge;2017;168–186.

[ref62] Walgrave S Swert K : The Making of the (Issues of the) Vlaams Blok. *Polit. Commun.* 2004;21:479–500.

[ref63] Weyland K : Clarifying a Contested Concept: Populism in the Study of Latin American Politics. *Comp. Polit.* 2001;34(1):1–22. New Brunswick: City University of New York.

[ref64] Zulianello M : Varieties of Populist Parties and Party Systems in Europe: From State-of-the-Art to the Application of a Novel Classification Scheme to 66 Parties in 33 Countries. *Government and opposition (London).* 2020;55(2):327–347. Cambridge: Cambridge University Press.

[ref65] Zulianello M Larsen EG : Blurred positions: The ideological ambiguity of valence populist parties. *Party Polit.* 2024;30(1):190–199. London, England: SAGE Publications.

