# Supplementary material for: Populism, euroscepticism, and the populism-euroscepticism nexus: a conceptual framework for analysis
Source: Open Res Eur. 2026 Jul 7;6:73. Originally published 2026 Mar 11. [Version 2] doi: 10.12688/openreseurope.23109.2 (PMC13560596; doi:10.12688/openreseurope.23109.2)
Supplement: Peer review history

# Reviewer report: Reviewer response for version 2

**DOI:** 10.21956/openreseurope.26432.r77120

**Published:** 2026-08-12

**Authors:** Kutsal Yesilkagit

**Affiliations:** 1 Institute of Public Administration, Leiden University Faculty of Governance and Global Affairs, The Hague, South Holland, The Netherlands

**Reviewer Report**

 This manuscript develops the conceptual framework for the EUPopLink project and discusses the relationship between populism and Euroscepticism in European party systems. I found the paper informative and useful in several respects. It provides a broad overview of the main literatures on both concepts, discusses different approaches to populism in some detail, and makes a serious attempt to formulate common definitions that can be used across a large number of country studies. I also appreciate that the authors do not simply assume that populism and Euroscepticism are the same phenomenon, and that they recognise considerable variation among populist parties in how they relate to European integration. 

 At the same time, I think that several conceptual issues need further clarification. My main concerns are about the derivation of the three types of populism, the distinction between populism and Euroscepticism, and the way in which the paper understands the political “supply side”. 

**1. The three categories of populism**

 It is not entirely clear to me how the authors arrive at the three categories of populism that they distinguish later in the paper: populist radical right, left-wing populism and valence populism. 

 The preceding discussion mainly deals with different approaches to defining populism: ideational, discursive, stylistic and strategic. The authors then settle on a definition of populism based on anti-elitism and popular sovereignty. That part is reasonably clear. What is less clear is how this discussion leads to the three categories that follow. 

 The populist radical right and left-wing populism are relatively easy to understand because populism is combined with a more recognisable ideological position. In the first case, this concerns nativism and authoritarianism; in the second case, a critique of capitalism and an emphasis on redistribution and alternative economic arrangements. 

 The category of “valence populism”, however, needs more explanation. It is described mainly in terms of issues such as corruption, transparency, democratic reform, moral integrity and anti-establishment politics. But these elements seem very close to the general people-versus-elites logic that the authors themselves see as central to populism. It is therefore not clear what exactly makes valence populism a distinct subtype. 

 The authors mention that this type became particularly important in Central and Eastern Europe and give examples such as  ANO and the Five Star Movement. These examples are useful, but they do not in themselves solve the conceptual problem. I would encourage the authors to explain more clearly how “the people” and “the elite” are constructed in valence populism, how popular sovereignty is expressed, and in what respect this category differs systematically from the other two forms. 

 More generally, the paper would benefit from a clearer explanation of how the three categories are derived from the general definition of populism, rather than simply introduced after the conceptual discussion. 

**2. Populism and Euroscepticism as analytically distinct concepts**

 My second concern is more fundamental. The paper treats populism and Euroscepticism as two analytically distinct concepts and then investigates their relationship. This is certainly possible, but I do not think the paper yet explains sufficiently why the two should be treated as separate concepts in the European context. 

 Euroscepticism emerged as a political phenomenon from the 1980s onwards, while contemporary European populism developed during roughly the same period. More importantly, European populism developed in a context in which European integration became increasingly important for domestic politics. This raises the question of whether populism and Euroscepticism are not partly intertwined from the outset. 

 The manuscript itself points in this direction. The EU can easily be presented in populist discourse as an elite-driven organisation, far removed from ordinary citizens and limiting the expression of popular sovereignty. “Brussels elites”, European bureaucrats or supranational institutions can therefore become part of the populist opposition between “the people” and “the elite”. 

 This makes it important to explain more clearly how the two concepts can be distinguished analytically. If opposition to EU elites forms part of a populist construction of the political conflict, when is this an expression of populism and when is it an expression of Euroscepticism? And when is it both? 

 This is also a historical question. Populism does not develop outside its political context. Latin American populism, for example, has been shaped by the specific historical and political context of Latin America, including post-colonialism, anti-imperialism and particular state-society relations. In a similar way, European populism has developed in a context in which European integration has become an important political cleavage and institutional constraint. 

 I therefore think the paper should discuss more explicitly whether contemporary European populism and Euroscepticism are simply two separate phenomena that frequently occur together, or whether they are partly constitutive of one another. 

 This does not mean that they cannot be separated. Clearly, non-populist Euroscepticism exists, and not every populist party is necessarily strongly Eurosceptic. But if the paper wants to study the “nexus” between the two concepts, it should make the conceptual boundary more explicit. 

 One way to do this would be to provide concrete examples of four different situations: populist but not Eurosceptic, Eurosceptic but not populist, both populist and Eurosceptic, and neither. This would make much clearer what exactly is being compared. 

**3. What is meant by the political “supply side”?**

 A third issue concerns the use of the term “supply side”. The paper states that it mainly focuses on the political supply side, but it could be clearer about what exactly is being supplied, and by whom. 

 In practice, political parties seem to be the main actors producing both populist discourse and Eurosceptic positions. If this is the case, then the same actors are being classified along two dimensions: first according to their degree or type of populism, and second according to their position towards European integration. 

 There is nothing wrong with this, but it would help if the analytical logic were stated more explicitly. What are the main sources of populist ideology on the one hand and Euroscepticism on the other? Are they generated by different ideological or political processes, or are they often produced by the same political parties and leaders? 

 This question becomes particularly relevant in cases where the two overlap. If a party claims that “Brussels elites” prevent “the people” from exercising their sovereignty, is this coded as populism, Euroscepticism, or both? The paper needs to explain more clearly how these dimensions are separated in empirical analysis. 

 I think it would help to distinguish between three things: the political actor supplying the message, the populist content of that message, and the position taken towards European integration. This would make the supply-side argument conceptually cleaner. 

**Overall assessment**

 I think this is a useful and potentially important conceptual paper, particularly because it provides the basis for a wider comparative research project. The literature review is broad, and the authors are clearly aware of the complexity of both concepts. 

 My main concern is that the paper currently moves somewhat too quickly from defining populism and Euroscepticism separately to analysing their relationship. Before doing so, the authors need to clarify more explicitly where the conceptual boundaries lie. In particular, I would encourage the authors to address the three points mentioned above.  

 Addressing these issues would considerably strengthen the conceptual foundations of the paper and, by extension, the comparative country studies that build on it.

Are all factual statements correct and adequately supported by citations?

Yes

Is the review written in accessible language?

Yes

Are the conclusions drawn appropriate in the context of the current research literature?

Partly

Is the topic of the review discussed comprehensively in the context of the current literature?

Yes

Reviewer Expertise:

Public administration; Democratic backsliding; political-administrative relationships; International governance

I confirm that I have read this submission and believe that I have an appropriate level of expertise to confirm that it is of an acceptable scientific standard, however I have significant reservations, as outlined above.

# Reviewer report: Reviewer response for version 2

**DOI:** 10.21956/openreseurope.26432.r77019

**Published:** 2026-08-08

**Authors:** Natasza Styczynska

**Affiliations:** 1 Jagiellonian University, Kraków, Poland

I appreciate the revisions that have been made and recommend the article for indexing.

Are all factual statements correct and adequately supported by citations?

Yes

Is the review written in accessible language?

Yes

Are the conclusions drawn appropriate in the context of the current research literature?

Partly

Is the topic of the review discussed comprehensively in the context of the current literature?

Yes

Reviewer Expertise:

Euroscepticism, Populism, Central and Eastern Europe, Western Balkans, EU integration, Enlargement

I confirm that I have read this submission and believe that I have an appropriate level of expertise to confirm that it is of an acceptable scientific standard.

# Reviewer report: Reviewer response for version 2

**DOI:** 10.21956/openreseurope.26432.r77018

**Published:** 2026-07-21

**Authors:** George Newth

**Affiliations:** 1 University of Bath, Bath, England, UK

My sincere thanks to the authors for addressing each suggestion so meticulously and in such good faith. I think this is an excellent paper, and I have no further comments or suggestions.

Are all factual statements correct and adequately supported by citations?

Yes

Is the review written in accessible language?

Yes

Are the conclusions drawn appropriate in the context of the current research literature?

Partly

Is the topic of the review discussed comprehensively in the context of the current literature?

Yes

Reviewer Expertise:

My research focuses, principally, on how populist, far right and nationalist forms of politics are normalised via discourses of “common sense”.

I confirm that I have read this submission and believe that I have an appropriate level of expertise to confirm that it is of an acceptable scientific standard.

# Reviewer report: Reviewer response for version 1

**DOI:** 10.21956/openreseurope.24999.r71063

**Published:** 2026-04-14

**Authors:** George Newth

**Affiliations:** 1 University of Bath, Bath, England, UK

This is an incredibly useful, thorough, and well-written overview of the literature on populism, Euroscepticism and the various links between the two phenomena. The authors are amongst the leading experts in the field and this is reflected in the comprehensive nature of the report. In a very busy field, the authors succeed in teasing out revised and accessible definitions of both populism and Euroscepticism, which I believe will be useful to not only academics and policy makers, but also the general public.

 I have listed below some minor suggestions which I hope might tighten up/clarify a few parts of the paper:

 I appreciate the skilful navigation of the minefield of different definitions of populism, and I think a certain centre of gravity is successfully found in Margaret Canovan’s approach. I do wonder, however, whether the difference between the ideational and discursive approach is somewhat downplayed in this process. For instance, the authors state

**both start out from the idea that the two central categories to populism (‘the people’ and ‘the elites’) are empty vessels that need to be given a more concrete interpretation (depending on political context and ‘host ideology’).**

 I would argue that there should be a bit more nuance here, if possible and if space permits. Does the thin-ideology approach not already provide some ideological content to both the people and the elites vis-à-vis framings of homogeneity and moralism (pure v corrupt)? In that respect can the people and the elites ever really be empty vessels in the ideational approach? By arguing that populists view all elites as corrupt and all people as ‘pure’, there is a clear belief system at work here. There is also something to be said about the fact that the homogeneity thesis favours analysis of the far right (see, Katsambekis 2022). On this point, I believe Jonathan Dean’s and Bice Maiguashca’s work on populism could be useful. Furthermore, these authors make a compelling argument for considering the stylistic approach as an ‘offshoot’ of the ideational approach insofar as it involves viewing populism as a ‘belief system’.  

 Refer to reference no. 1

 A further issue, is raised in the statement below:

**We also concur with the notion that populism can be measured at the political supply side (i.e. expressed by political actors) as well as the political demand side (i.e. as an attitude of citizens)**

 The idea of the ‘political demand’ is always a bit problematic and might need a footnote to indicate the nuance of ‘public opinion’ construction via top-down (and often skewed) surveys. Seongchul Kim’s and Aurelien Mondon’s work on ‘populist attitudes’ could be useful here insofar as they illustrate that the attitudes approach in its reliance on opinion polls risks treating public opinion as a fixed reality and assuming that “the people” have clear, objective qualities.

 Refer to reference no. 2

 More generally, there is emergent literature that populism is not something which is at all alien to Europe/European values, but which is at the heart of European politics. While I realise this is not the correct forum for lengthy debates around this, I wonder whether this could at least be acknowledged, not least because the authors draw the correct distinction between the EU and Europe. See, for instance, not only recent literature from Emmy Eklundh on Europe’s populist condition/coloniality, but also from Alex Yates on European Values and anti-populism.

 In terms of Euroscepticism, the contingent nature of this identity/discourse can also be measured by the fact that some parties change their position as they evolve. The Lega (Nord), for instance, was not Eurosceptic to begin with, but took a relatively Euro-pragmatist position in line with the idea of a ‘Europe of the Regions’. It maintained this position between 1992 and 1998 (Maccaferri and Newth 2022). Conversely, some parties start with *just *Euroscepticism as their key raison d’être e.g. AfD and UKIP (originally the anti-federalist league), and then shift to being part of the populist radical right by adopting increasingly reactionary positions.  Is there a way of integrating this element of historical contingency into the report, albeit briefly?

 Regarding the comment that:

**Meloni enjoys seemingly cozy and constructive relationships with various European leaders, illustrating the continuing legitimisation and normalisation of the far right (** **Mudde, 2019** **).**

 I would ask the question of whether this is just a softening of Meloni’s image or also a radicalization of the EU in terms of, for instance, their Asylum and Migration policy which makes it easier for far right figures to claim the EU is adopting its policies. (again this goes back to Eklundh’s work on ‘Europe’s populist condition’, and the fact that such anti-migrant/nativist/racist policies are not at all alien to Europe or European values).

 The mention of transnationalism in the final paragraphs of the populism-Europscepticism nexus is welcome, but I wonder whether mentioning Diem25 would make sense here as a clearer and more explicit example. This is a potentially good example of a leftist movement which manages to straddle both opposition to certain aspects of the EU, while taking a ‘pan-European’ approach. The fact that the party also failed to elect any candidates, might also speak to the limitations in such an approach.

 One further minor shortcoming in the third section is the lack of development of the issue of Valence Populism. This, I would argue is quite important, considering that one of the key 'Valence Populist' actors in the Five Star Movement, has had evolving views on Euroscepticism (and migration), see for instance:

 Refer to reference no. 3

 A further point is that there does appear to be a clear regional bias in the case studies presented. This is not necessarily a major issue,  as the authors use the case studies very effectively. However, I would argue that the slightly western-centric nature of the examples/case studies should be acknowledged/justified from the outset.  

 A couple of more minor points relate to terminological consistency which I think  could be confusing to some readers and risks undermining the careful definitional work previously established by the authors. For instance in the third section, the authors state

**‘A second variant of populism, the populism-nativist discourse, delineates ‘the people’ along nationalistic and identity-based lines ( Lobera and Roch, 2022).’**

 In what ways is 'populism-nativism' different to the Populist Radical Right, which the authors have carefully outlined in the section on populism?

 Similarly, the term  ‘radical populist parties’ in this section is a bit unclear; this could mean either Populist Radical Right, or left wing populism, in theory.

 Overall, I think this is a very good piece and I really appreciate the careful, meticulous research involved in putting it together. I hope my comments are useful, relatively minor, and not too difficult to address

Are all factual statements correct and adequately supported by citations?

Yes

Is the review written in accessible language?

Yes

Are the conclusions drawn appropriate in the context of the current research literature?

Partly

Is the topic of the review discussed comprehensively in the context of the current literature?

Yes

Reviewer Expertise:

My research focuses, principally, on how populist, far right and nationalist forms of politics are normalised via discourses of “common sense”.

I confirm that I have read this submission and believe that I have an appropriate level of expertise to confirm that it is of an acceptable scientific standard, however I have significant reservations, as outlined above.

## References

[1] : Did somebody say populism? Towards a renewal and reorientation of populism studies. *Journal of Political Ideologies* .2020;25(1) : 10.1080/13569317.2020.169971211-27 10.1080/13569317.2020.1699712

[2] : From Objectivist Bias to Positivist Bias: A Constructivist Critique of the Attitudes Approach to Populism. *Political Studies Review* .2024;22(4) : 10.1177/14789299231225403985-999 10.1177/14789299231225403

[3] : At the origins of the political discourse of the 5-Star Movement (M5S): Internet, direct democracy and the “future of the past”. *Internet Histories* .2018;2(1-2) : 10.1080/24701475.2018.145729598-120 10.1080/24701475.2018.1457295

# Response

**Published:** 2026-06-22

**Authors:** Juan Roch

**Affiliations:** Political Science and International Relations, Autonomous University of Madrid Department of Political Science and International Relations, Madrid, Community of Madrid, Spain

Dear **George Newth**,   Many thanks for the tone of your review and for your very useful comments. We respond to each point below and explain the revisions we have made to the manuscript in light of your suggestions.  

*1* *.* *I would argue that there should be a bit more nuance here, if possible and if space permits. Does the thin-ideology approach not already provide some ideological content to both the people and the elites vis-à-vis framings of homogeneity and moralism (pure v corrupt)? In that respect can the people and the elites ever really be empty vessels in the ideational approach? By arguing that populists view all elites as corrupt and all people as ‘pure’, there is a clear belief system at work here. There is also something to be said about the fact that the homogeneity thesis favours analysis of the far right (see, Katsambekis 2022). On this point, I believe Jonathan Dean’s and Bice Maiguashca’s work on populism could be useful. Furthermore, these authors make a compelling argument for considering the stylistic approach as an ‘offshoot’ of the ideational approach insofar as it involves viewing populism as a ‘belief system’.* *Refer to reference no. 1*

 Thank you for this comment. We have clarified this point in the revised manuscript. We agree that the expression “empty vessels” is more appropriate for the discursive approach than for the ideational one, even though the latter conceptualizes populism as a thin-centred ideology in which ‘the people’ and ‘the elites’ remain relatively vague categories that allow for considerable ideological specification through different host ideologies. Accordingly, we have reformulated the relevant passage and narrowed the common ground between the ideational and discursive approaches to the idea that ‘the people’ and ‘the elites’ function as relatively flexible categories that are open to a variety of ideological and political articulations. Moreover, in the preceding paragraph, we now further develop the distinction between the two approaches and clarify the implications of defining ‘the people’ as a homogeneous entity, including its tendency to privilege research on far-right populism.  

*2. A further issue, is raised in the statement below:* ***We also concur with the notion that populism can be measured at the political supply side (i.e. expressed by political actors) as well as the political demand side (i.e. as an attitude of citizens)***** *The idea of the ‘political demand’ is always a bit problematic and might need a footnote to indicate the nuance of ‘public opinion’ construction via top-down (and often skewed) surveys. Seongchul Kim’s and Aurelien Mondon’s work on ‘populist attitudes’ could be useful here insofar as they illustrate that the attitudes approach in its reliance on opinion polls risks treating public opinion as a fixed reality and assuming that “the people” have clear, objective qualities.* *Refer to reference no. 2*

 Thank you for this comment. We have now addressed this issue in the manuscript by referring to the ongoing debate surrounding the measurement of populist attitudes. We also refer to the thought-provoking critique advanced by Kim and Mondon (2024).

*3. More generally, there is emergent literature that populism is not something which is at all alien to Europe/European values, but which is at the heart of European politics. While I realise this is not the correct forum for lengthy debates around this, I wonder whether this could at least be acknowledged, not least because the authors draw the correct distinction between the EU and Europe. See, for instance, not only recent literature from Emmy Eklundh on Europe’s populist condition/coloniality, but also from Alex Yates on European Values and anti-populism.*

 We consider these to be important contributions, and we have therefore included a short paragraph discussing the relationship between the construction of Europe, the European Union, and populism. We hope this addition broadens the scope of the article.

*4.In terms of Euroscepticism, the contingent nature of this identity/discourse can also be measured by the fact that some parties change their position as they evolve. The Lega (Nord), for instance, was not Eurosceptic to begin with, but took a relatively Euro-pragmatist position in line with the idea of a ‘Europe of the Regions’. It maintained this position between 1992 and 1998 (Maccaferri and Newth 2022). Conversely, some parties start with just Euroscepticism as their key raison d’être e.g. AfD and UKIP (originally the anti-federalist league), and then shift to being part of the populist radical right by adopting increasingly reactionary positions.  Is there a way of integrating this element of historical contingency into the report, albeit briefly?*

 Thank you for your suggestion. In response to this comment, we expanded the section on the contingent nature of Euroscepticism. We have added a few examples of parties that switched positions from support to opposition and vice-versa. We have also included a point on formerly single-issue anti-EU parties giving the European issue less salience.  

 5. Regarding the comment that:   Meloni enjoys seemingly cozy and constructive relationships with various European leaders, illustrating the continuing legitimisation and normalisation of the far right (Mudde, 2019).   I would ask the question of whether this is just a softening of Meloni’s image or also a radicalization of the EU in terms of, for instance, their Asylum and Migration policy which makes it easier for far right figures to claim the EU is adopting its policies. (again this goes back to Eklundh’s work on ‘Europe’s populist condition’, and the fact that such anti-migrant/nativist/racist policies are not at all alien to Europe or European values). 

 Thank you for this comment. We agree that this dynamic is more appropriately described as a reciprocal and two-sided relationship rather than simply as a process of moderation on the part of PRR parties. We have clarified this point in the revised manuscript (p. 12).

   6. The mention of transnationalism in the final paragraphs of the populism-Europscepticism nexus is welcome, but I wonder whether mentioning Diem25 would make sense here as a clearer and more explicit example. This is a potentially good example of a leftist movement which manages to straddle both opposition to certain aspects of the EU, while taking a ‘pan-European’ approach. The fact that the party also failed to elect any candidates, might also speak to the limitations in such an approach. 

 Thank you for this suggestion. We agree that DIEM25 provides an illuminating example of (ultimately unsuccessful) transnational populism. We have therefore included a reference to the movement, as well as to recent research examining the factors that contributed to its failure (Oleart and Weisskircher, 2025).  

 7. One further minor shortcoming in the third section is the lack of development of the issue of Valence Populism. This, I would argue is quite important, considering that one of the key 'Valence Populist' actors in the Five Star Movement, has had evolving views on Euroscepticism (and migration), see for instance:   Refer to reference no. 3   Thank you for this comment. We have clarified that certain critiques of the EU stem from populism itself and can therefore be found across different variants of populism. At the same time, we now make more explicit that ideological content becomes relevant when explaining the specific form these critiques take, a distinction that primarily concerns right-wing and left-wing populism rather than valence populism.  

 8. A further point is that there does appear to be a clear regional bias in the case studies presented. This is not necessarily a major issue,  as the authors use the case studies very effectively. However, I would argue that the slightly western-centric nature of the examples/case studies should be acknowledged/justified from the outset.    

  Thank you for this comment. We agree with this observation and have incorporated additional examples and reflections on Central and Eastern European countries throughout the manuscript.  

 9. A couple of more minor points relate to terminological consistency which I think could be confusing to some readers and risks undermining the careful definitional work previously established by the authors. For instance in the third section, the authors state  ‘A second variant of populism, the populism-nativist discourse, delineates ‘the people’ along nationalistic and identity-based lines (Lobera and Roch, 2022).’  In what ways is 'populism-nativism' different to the Populist Radical Right, which the authors have carefully outlined in the section on populism?  Similarly, the term ‘radical populist parties’ in this section is a bit unclear; this could mean either Populist Radical Right-, or left-wing populism, in theory.  

  We agree with this observation and have revised these formulations to improve their accuracy and clarity throughout the manuscript.   Overall, I think this is a very good piece and I really appreciate the careful, meticulous research involved in putting it together. I hope my comments are useful, relatively minor, and not too difficult to address

# Reviewer report: Reviewer response for version 1

**DOI:** 10.21956/openreseurope.24999.r71057

**Published:** 2026-04-08

**Authors:** Natasza Styczynska

**Affiliations:** 1 Jagiellonian University, Kraków, Poland

The article provides a conceptual framework for studying the populism–Euroscepticism nexus within COST action 'Linking euroscepticism and populism: causes and consequences' (EUPopLink).

 The article is well structured and easy to follow. It begins by exploring the concept of populism before moving on to examine Euroscepticism. It then goes on to analyse the links between the two. While the study is theoretically robust, its focus on Western European examples may give the impression that other regions are either unaffected by the phenomenon or have received less research attention. However, this is not the case.

 The first part of the discussion focuses on the various definitions of populism. The authors have chosen to concentrate on the supply-side of populism and Euroscepticism. I am curious as to the reasoning behind this. It would be beneficial to understand why authors only focus on the supply side, while the demand side also tells us about the nexus between these two phenomena.

 The discussion presents an ideational and discursive approach. For the needs of the COST project, populism will be understood as a dichotomy between the people and the elite, and three types of populism: right-wing, left-wing and value populism will be studied.

 The authors observe that valence populism 'has been particularly widespread in Central and Eastern Europe', a statement that is open to debate. While it is accurate for some of the first populist movements and parties, and can represent the evolution of particular cases, such as the Hungarian Fidesz party, it would not prove to be valid when analysing the value-driven populist arguments, especially of the actors characterised also by the Eurosceptic stance (and those are a primary focus of the COST network). The CEE is not a coherent region, which may make it challenging to generalise.

 The following section, focused on Euroscepticism, is also firmly rooted in the academic discourse, underscoring the key concerns surrounding the term and highlighting the primary categorisations that have been established over time. The project builds on Paul Taggart's definition of Euroscepticism. It will analyse the object, nature and intensity of the opposition to European integration.

 Following a thorough conceptualisation of both phenomena, the article goes on to explore the connection between populism and Euroscepticism.

 While the section on conceptualisation of populism distinguishes 'valence populism', the section exploring the populism-Euroscepticism nexus does not engage with this category, focusing instead on left and right-wing populism only.

 Except for a mention of the Hungarian Fidesz party, the provided examples cover populist and Eurosceptic actors in Western and Southern Europe. Surprisingly, the paper does not engage with cases from Nordic countries, Central, Eastern and South-Eastern Europe. Cases such as EKRE, Konferderacja, SNS, Sweden Democrats, Smer, Vazrazhdane and Romanian AUR, to name but a few, are of significance and could serve to demonstrate the phenomenon of populist Euroscepticism. There is a rich body of existing literature on these parties/countries and including it would enhance the paper and provide the reader with a more comprehensive understanding of the European populist-Euroscepticism.

 In summary, this is a well-written and well-researched article that provides a solid foundation for further research. However, two issues have led to some reservations. Firstly, there is a lack of connection between valence populism and populist Euroscepticism in the section on the nexus between the two. Secondly, it should be noted that the geographical bias of the paper is notable, as it is based mostly on Western examples, while the COST network covers all of Europe, and the country reports cover 40 countries.

Are all factual statements correct and adequately supported by citations?

Yes

Is the review written in accessible language?

Yes

Are the conclusions drawn appropriate in the context of the current research literature?

Partly

Is the topic of the review discussed comprehensively in the context of the current literature?

Yes

Reviewer Expertise:

Euroscepticism, Populism, Central and Eastern Europe, Western Balkans, EU integration, Enlargement

I confirm that I have read this submission and believe that I have an appropriate level of expertise to confirm that it is of an acceptable scientific standard, however I have significant reservations, as outlined above.

# Response

**Published:** 2026-06-22

**Authors:** Juan Roch

**Affiliations:** Political Science and International Relations, Autonomous University of Madrid Department of Political Science and International Relations, Madrid, Community of Madrid, Spain

Dear Natasza Styczynska,

  Thank you very much for your constructive and insightful comments. We respond to each point below and outline the revisions we have made in the revised version of the manuscript.

*1. The article provides a conceptual framework for studying the populism–Euroscepticism nexus within COST action 'Linking euroscepticism and populism: causes and consequences' (EUPopLink).* *The article is well structured and easy to follow. It begins by exploring the concept of populism before moving on to examine Euroscepticism. It then goes on to analyse the links between the two. While the study is theoretically robust, its focus on Western European examples may give the impression that other regions are either unaffected by the phenomenon or have received less research attention. However, this is not the case.*

**Thanks for the positive comments, and also for pointing out that the use of examples can be drawn from a wider geographical area. We sought to address this by referring to more non-WE cases as illustrative examples. Note that this text primarily serves as a conceptual framework and basis for the country reports in this collection – so it does not aim to provide a systematic analysis of populist Euroscepticism, and empirical examples are mainly used to clarify concepts and theory.  

*2. The first part of the discussion focuses on the various definitions of populism. The authors have chosen to concentrate on the supply-side of populism and Euroscepticism. I am curious as to the reasoning behind this. It would be beneficial to understand why authors only focus on the supply side, while the demand side also tells us about the nexus between these two phenomena.*

 The reviewer is completely right to point this out, and the demand side will certainly also be covered by the broader EUPopLink project. But, beyond general conceptualization, our WG1 was primarily tasked with mapping populism and Euroscepticism on the supply side, and this article serves as a foundational article for the country reports that are also part of this ORE collection. We have clarified this in the introduction

*3. The discussion presents an ideational and discursive approach. For the needs of the COST project, populism will be understood as a dichotomy between the people and the elite, and three types of populism: right-wing, left-wing and value populism will be studied.* *The authors observe that valence populism 'has been particularly widespread in Central and Eastern Europe', a statement that is open to debate. While it is accurate for some of the first populist movements and parties, and can represent the evolution of particular cases, such as the Hungarian Fidesz party, it would not prove to be valid when analysing the value-driven populist arguments, especially of the actors characterised also by the Eurosceptic stance (and those are a primary focus of the COST network). The CEE is not a coherent region, which may make it challenging to generalise.*

**The reviewer is right that this passage was too crude and lacked nuance. The revised text attempts to clarify the specific time span of these examples and to avoid generalization. Some more (recent) examples from CEE countries are integrated in the remainder of the text. 

*4. The following section, focused on Euroscepticism, is also firmly rooted in the academic discourse, underscoring the key concerns surrounding the term and highlighting the primary categorisations that have been established over time. The project builds on Paul Taggart's definition of Euroscepticism. It will analyse the object, nature and intensity of the opposition to European integration.* *Following a thorough conceptualisation of both phenomena, the article goes on to explore the connection between populism and Euroscepticism.* *While the section on conceptualisation of populism distinguishes 'valence populism', the section exploring the populism-Euroscepticism nexus does not engage with this category, focusing instead on left and right-wing populism only.*

 Two amendments in the section now clarify that all types of populism (left, right, valence) are likely to share certain critiques of the EU (those that are related to their populism per se). Where we discuss the additional role of ideological leaning for the substantive content and arguments forming the EU critique of political actors, we clarify that here only the PRR and left-wing populist variants are relevant, given that ‘valence populism’ is not inherently ideologically oriented.

*5. Except for a mention of the Hungarian Fidesz party, the provided examples cover populist and Eurosceptic actors in Western and Southern Europe. Surprisingly, the paper does not engage with cases from Nordic countries, Central, Eastern and South-Eastern Europe. Cases such as EKRE, Konferderacja, SNS, Sweden Democrats, Smer, Vazrazhdane and Romanian AUR, to name but a few, are of significance and could serve to demonstrate the phenomenon of populist Euroscepticism. There is a rich body of existing literature on these parties/countries and including it would enhance the paper and provide the reader with a more comprehensive understanding of the European populist-Euroscepticism.*

 We agree with the reviewer that the use of examples can be drawn from a wider geographical area. We sought to address this by referring to several more non-WE cases as illustrative examples at various places in the article. We also added a paragraph that highlights the wide geographical scope of the project (that also covers candidate countries). Note, however, that this text primarily serves as a conceptual framework and basis for the country reports in this collection – so it does not aim to provide a systematic analysis of populist Euroscepticism, and empirical examples are mainly used to clarify concepts and theory. This is also explained now at the start of the ‘nexus’ section.  The rich country-specific empirical material can be found in the country reports that are part of this ORE collection.

*In summary, this is a well-written and well-researched article that provides a solid foundation for further research. However, two issues have led to some reservations. Firstly, there is a lack of connection between valence populism and populist Euroscepticism in the section on the nexus between the two. Secondly, it should be noted that the geographical bias of the paper is notable, as it is based mostly on Western examples, while the COST network covers all of Europe, and the country reports cover 40 countries.*
